# Homozygosity and risk of childhood death due to invasive bacterial disease

**DOI:** 10.1186/1471-2350-10-55

**Published:** 2009-06-12

**Authors:** Emily J Lyons, William Amos, James A Berkley, Isaiah Mwangi, Mohammed Shafi, Thomas N Williams, Charles R Newton, Norbert Peshu, Kevin Marsh, J Anthony G Scott, Adrian VS Hill

**Affiliations:** 1 The Wellcome Trust Centre for Human Genetics, University of Oxford, Roosevelt Drive, Oxford OX3 7BN, UK; 2 Department of Zoology, University of Cambridge, Downing Street, Cambridge CB2 3EJ, UK; 3 Centre for Geographic Medicine Research (Coast), Kilifi, Kenya; 4 Current address: MRC Centre for Outbreak Analysis and Modeling, Department of Infectious Disease Epidemiology, Imperial College, St. Mary's Campus, Norfolk Place, London W2 1PG, UK

## Abstract

**Background:**

Genetic heterozygosity is increasingly being shown to be a key predictor of fitness in natural populations, both through inbreeding depression, inbred individuals having low heterozygosity, and also through chance linkage between a marker and a gene under balancing selection. One important component of fitness that is often highlighted is resistance to parasites and other pathogens. However, the significance of equivalent loci in human populations remains unclear. Consequently, we performed a case-control study of fatal invasive bacterial disease in Kenyan children using a genome-wide screen with microsatellite markers.

**Methods:**

148 cases, comprising children aged <13 years who died of invasive bacterial disease, (variously, bacteraemia, bacterial meningitis or neonatal sepsis) and 137 age-matched, healthy children were sampled in a prospective study conducted at Kilifi District Hospital, Kenya. Samples were genotyped for 134 microsatellite markers using the ABI LD20 marker set and analysed for an association between homozygosity and mortality.

**Results:**

At five markers homozygosity was strongly associated with mortality (odds ratio range 4.7 – 12.2) with evidence of interactions between some markers. Mortality was associated with different non-overlapping marker groups in Gram positive and Gram negative bacterial disease. Homozygosity at susceptibility markers was common (prevalence 19–49%) and, with the large effect sizes, this suggests that bacterial disease mortality may be strongly genetically determined.

**Conclusion:**

Balanced polymorphisms appear to be more widespread in humans than previously appreciated and play a critical role in modulating susceptibility to infectious disease. The effect sizes we report, coupled with the stochasticity of exposure to pathogens suggests that infection and mortality are far from random due to a strong genetic basis.

## Background

Many recent studies of natural populations report a correlation between genetic heterozygosity (heterozygosity-fitness correlation, HFC), measured at a small number (of the order of 10) of presumed neutral markers, and fitness [1,2]. Fitness measures range widely from survival [3] and reproductive success [4-6] to indirect traits such as song complexity [7] and territory size [8] in birds. Some of the most frequently reported traits relate to immune function [9] and susceptibility to micro- [10] and macroparasites [11-13]. Such studies raise obvious questions, both about the mechanism responsible, and whether similar patterns may affect humans.

Two primary mechanisms have been suggested to explain HFCs [14,15]. First, relatively homozygous individuals may be more susceptible to infection because they are inbred. Here, average heterozygosity at the panel of markers being genotyped estimates genome-wide heterozygosity, which in turn estimates the inbreeding coefficient, *F*. However, several theoretical treatments have come to the conclusion that such a mechanism is unlikely to operate in most real populations [16-18]. The problem is that random mating generates extremely few individuals with sufficiently high *F *for their heterozygosity to stand out when measured at tens or even hundreds of markers, unless the population is very small or highly polygynous. Humans may offer a further exception in cultures where cousin marriages are actively encouraged [19], potentially increasing the rate of heritable diseases [20,21].

The second mechanism that may generate HFCs involves chance linkage between one or more of the markers and a gene(s) experiencing balancing selection. Balancing selection has often been thought to be rather rare, particularly in humans [22] where the classical example is sickle cell anemia [23] remains one of very few examples. Moreover, while some argue that polymorphism at immune function genes is maintained by overdominant balancing selection [24], there is evidence that this is unlikely to be effective at maintaining more than two alleles [25-27]. Regardless of theory, a number of recent HFC studies report convincing associations between heterozygosity at one particular locus and the measured trait [13,28-31].

Over the last five to ten years, association studies examining the genetic basis of human disease have switched overwhelmingly from microsatellite markers to single nucleotide polymorphisms (SNPs) [32]. SNPs are much less polymorphic than microsatellites, a deficiency that is usually compensated for by the vastly greater number of markers being genotyped. However, while there are many advantages to using SNPs for the assessment of local heterozygosity, microsatellites offer an arguably more direct approach that circumvents the need to reconstruct complex haplotypes. To assess the possible importance of HFCs in humans, we therefore conducted a case-control study in a population of Kenyan children, using a panel of microsatellite markers to quantify both local and genome-wide heterozygosity.

## Results

All our samples were drawn from a prospective study in Kilifi District Hospital and were genotyped for 134 microsatellite markers using the ABI LD20 marker set (Applied Biosystems, USA) [see Additional file 1]. Cases (n = 148) comprised a consecutive series of children aged <13 years who died of invasive bacterial disease, (variously, bacteraemia, bacterial meningitis or neonatal sepsis, for details see methods), a major contributor to childhood mortality in the developing world [33]. Controls comprised 137 randomly selected healthy children matched on age to the cases. Microsatellite traces were scrutinised carefully to ensure homozygotes were identified with high accuracy.

For the study of HFCs a number of measures of heterozygosity have been proposed that offer potential benefits over straight heterozygosity, weighting scores variously by allele size (mean d^2^)[3], allele frequencies (internal relatedness, IR) [6] and the variability of loci scored (HL) [34]. However, in automated high throughput studies, heterozygosity assessment can sometimes be problematic, particularly where time for scrutiny of every trace is limited. Thus, null (non-amplifying) alleles, allele drop-out and, at some loci, high levels of stutter-bands can all contribute to a tendency for a minority of loci to carry misleading genotypes where heterozygotes are called as homozygotes or vice versa. Issues have also been identified with allele binning, in some cases causing single alleles to be split between two length classes [35]. In an attempt to circumvent these problems we spent most empirical effort ensuring that heterozygotes and homozygotes were accurately scored and introduce a variant of the measure Standardised Heterozygosity (SH) [2], designed to be highly conservative. SH controls for missing data by expressing heterozygosity as the ratio of the observed heterozygosity in an individual relative to the expected value at the markers genotyped, assuming Hardy-Weinberg equilibrium. Our measure, Standardised Observed Homozygosity (SOH), follows the same principle but instead of calculating the expected homozygosity from the allele frequencies, we used the observed homozygosity at each locus. In this way, SOH measures the extent to which any given individual is more or less homozygous relative to the level expected if all genotypes were randomized among individuals, negating the requirement for accurate allele frequency estimates and reducing the impact of allele drop out, null alleles and other possible artefacts.

**Figure 1:**
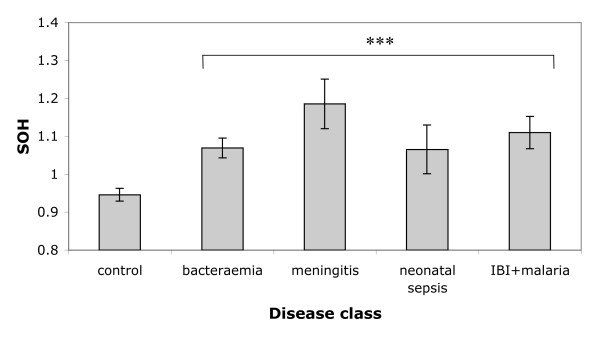
**Analysis of variance of standardized observed homozygosity values for cases and controls**. *S*_*OH *_is the Standardised Observed Homozygosity for an individual genotyped for *i *loci, calculated as:  where *N*_*hom *_is the number of homozygote genotypes in the individual concerned and *H*_*oi *_is the observed frequency of homozygotes at one of the *i *loci scored in this individual. ***indicates a highly significant test where P < 1 × 10^-5^. The IBI + malaria group includes individuals who had invasive bacterial disease but also malaria parasitaemia so that the contribution of the latter to mortality could not be determined with certainty. Sample sizes for the disease classes are as follows: control = 183, bacteraemia = 71, meningitis = 18, neonatal sepsis = 26 and IBI + malaria parasitaemia = 34. IBI: invasive bacterial infection. Error bars are ± 1 standard error.

We first asked whether SOH varied significantly among disease categories by conducting a one-way ANOVA. Raw SOH values exhibit a slightly skewed distribution, but this is removed by a simple log transformation (Shapiro-Wilk normality test, W = 0.9941, p = 0.322). Following transformation, SOH revealed highly significant variation among disease classes (F_ [5,281] _= 6.75, P = 5.89 × 10^-6^) (Figure 1). However, when the control class was excluded, the ANOVA was no longer significant (F _ [4,144] _= 0.785, P = 0.54), indicating that the main effect is driven by a difference in heterozygosity between cases and controls rather than between disease classes. The direction of the deviation is toward greater homozygosity in cases compared with the controls.

**Figure 2:**
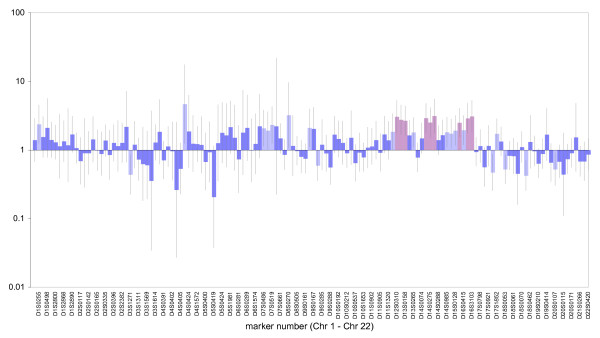
**Odds ratios and 95% confidence intervals for mortality and homozygosity by marker, adjusting for age**. Age-adjusted odds ratios (with 95% confidence intervals) of mortality at each locus. All markers were tested for significance using a chi-squared test based on a simple 2 × 2 contingency table (case/control vs homozygotes/heterozygotes). ORs shown in dark blue are non-significant. ORs shown in pale blue are significant at P < 0.05 and ORs shown in pink (n = 9) are significant at P < 0.00037 (i.e. significant experiment-wide at P < 0.05).

We next asked whether there was evidence of local effects due to chance linkage between one or more markers and a gene(s) experiencing balancing selection. To test this proposition we calculated age-adjusted odds ratios of mortality at each locus in turn (Figure 2). Most markers show either a non-significant or borderline (at alpha = 0.05) association between homozygosity and risk of mortality. However, nine markers reveal a strong associations with experiment wide significance using full Bonferroni correction (p < 0.00037, see Table 1). This is a highly conservative threshold since where multiple markers are expected, the less stringent false discovery rate approach can be justified [36]. Although the spacing between markers is sufficient to ensure they behave as if unlinked, it is possible that multiple markers contribute to the same risk through linkage to related genes. Consequently, we then constructed a multivariable logistic regression model with mortality as the response and age, sex, locality, SOH and homozygosity at each of the nine largest-effect markers as explanatory variables. Sequential removal of terms that did not contribute significantly (likelihood ratio test, p < 0.05) yielded a final model containing age, location and five markers (D12S310, D13S158, D14S275, D16S3103, D16S423). SOH is dropped as a marginally significant term (LHR, p~0.07) whether fitted as a continuous variable or as a factor with five levels. By implication, it seems that genome-wide effects (inbreeding depression) are minimal or absent.

**Table 1:** Nine microsatellites showing the strongest association between heterozygosity and mortality due to invasive bacterial disease.

**Marker**	**OR**	**lower 95% CI**	**upper 95% CI**	**chi sq**	**p value**
D12S310	2.86	1.68	4.86	15.420	0.000086

D12S352	2.66	1.64	4.31	16.194	0.000057

D13158	2.67	1.66	4.3	16.496	0.000049

D14S261	3.14	1.94	5.09	21.998	0.000003

D14S275	2.59	1.59	4.22	15.034	0.000106

D14S280	3.57	2.05	6.21	21.421	0.000004

D15S1007	2.64	1.63	4.28	16.093	0.000060

D16S423	2.60	1.6	4.21	15.368	0.000088

D16S3103	2.89	1.75	4.77	17.611	0.000027

The table shows odds ratios (OR), adjusted for age in 6 strata and geographical location in 8 strata, of death due to bacterial invasive disease, along with lower and upper 95% confidence intervals (CI). Significance was assessed using 2 × 2 contingency table of heterozygote/homozygote vs case/control, yielding a chi-squared value (chi sq) and its associated P value. All loci comfortably exceed the experiment-wide significance threshold of 0.00037.

Beginning with the final model derived above, we explored further possible interactions between markers, and also between each marker and age. Among all possible pair-wise combinations of markers, two revealed significant interactions, both of which were retained in the model regardless of the order in which they were added (Table 2, last two columns). No significant interactions with age were detected. Our data contain approximately equal numbers of individuals who died from Gram positive (n = 63), gram negative (n = 79) or both (n = 6) infections and this allowed us to ask whether our markers identify genes that impact differently on diseases caused by bacteria of different classes. We therefore repeated the logistic regression approach above on each bacterial class separately, including dual infections in both analyses. Given the smaller datasets, criteria for initial inclusion in the model were relaxed to an initial OR significant at p < 0.005. The final models are summarized in Table 2 and reveal surprising complexity, with susceptibility to gram positive and gram negative infections associated mostly with non-overlapping genomic locations. Only marker D12S310 is significant in all three models. Marker D9S164 reveals an interaction with age, infants being more likely to die if homozygous (OR = 1.65) and older children less (OR = 0.18). Interactions between marker pairs in the whole dataset suggest that homozygosity at both markers together confers no greater risk than homozygosity at either one alone. However, in the gram negative model the interaction of homozygosity at two markers, D7S486 and D16S423, indicates a significantly synergistic risk of mortality (odds ratio 40.7) where homozygosity at either of the markers alone confers no risk.

**Table 2:** Age- and geographic location-adjusted odds ratios for invasive bacterial death with homozygosity at specific microsatellite markers in multivariable models restricted to cases of Gram positive sepsis, gram negative sepsis or including all invasive bacterial deaths combined.

		**Gram Negative**		**Gram Positive**		**Combined**	
*Markers*	*Interaction*	*OR*	*95% CI*	*OR*	*95%CI*	*OR*	*95% CI*

D7S486		1.02	0.16 – 6.53				
D7S486	D16S423	40.7	4.28 – 387				
D12S310		14.0	2.70 – 72.7	4.73	1.59–14.1	4.94	2.27 – 10.8
D13S158		6.11	1.45 – 25.8			4.66	1.92 – 11.3
D15S1007		7.28	1.89 – 28.1				
D16S423		1.61	0.31 – 8.33			7.65	2.55 – 22.9
D9S164	infants			0.18	0.018 – 1.90		
D9S164	children			1.65	0.249 – 10.9		
D14S275				3.93	1.35 – 11.4	12.2	4.44 – 33.3
D14S275	D13S158					10.1	3.84 – 26.5
D16S3103				3.70	1.20 – 11.4	7.04	2.56 – 19.4
D16S3103	D16S423					10.2	4.10 – 25.2

The table shows odds ratios, adjusted for age in 6 strata and for geographical location in 8 strata, of death due to all invasive bacterial disease, of death due to Gram negative invasive bacterial disease and of death due to Gram positive invasive bacterial disease, for homozygosity at microsatellite markers either alone or paired (where interactions were noted with LRT p values < 0.05). Variables excluded, with LRT p values = 0.05, in addition to sex and SOH, were markers d12s352, d14s261, d14s275, d16s3103 and d18s452 in the gram negative model, markers d13s158, d14s261, d14s280 and d16s423 in the gram positive model and markers d12s352, d14s261, d14s280 and d15s1007 in the combined model. Variables and interaction terms in the final models all had LRT p values < 0.02.

Finally, to assess the magnitude of homozygosity effects with respect to the population, we calculated the population attributable risk fraction (PARF) [37] for each marker (Table 3). PARFs indicate the proportional reduction in mortality due to bacterial infection that would result if homozygosity at the locus could be eliminated. However, such direct interpretation in our case is problematic for many reasons, including the fact that the risk is probably driven by specific alleles whose prevalence differs greatly from that of homozygotes in general. None the less, the ORs of the full model indicate that the risks we describe are sizeable, particularly since the markers provide only indirect measures of homozygosity at the genes themselves. Furthermore, given that the population prevalence of homozygosity at the relevant markers is high, the population-wide effects of homozygosity are likely to impact very considerable on the total burden of invasive bacterial disease mortality, with the majority of deaths being genetically determined.

**Table 3:** Population attributable risk fractions (PARF) for homozygosity at five microsatellite markers in a final multivariable model of bacterial diseases death.

**Microsatellite marker**	**population prevalence of homozygosity**	**OR for bacterial disease death**	**PARF**
D12S310	0.187	3.65	0.331
D13S158	0.387	2.04	0.287
D14S275	0.282	4.68	0.509
D16S3103	0.490	3.52	0.553
D16S423	0.418	2.75	0.423

PARFs were calculated using all markers retained in the final multivariable logistic regression model but, for simplicity, without interaction effects. For this reason the Odds Ratios quoted here are not identical to those in table 2.

## Discussion

Here we conduct what we believe is the first systematic analysis of the association between heterozygosity and infectious diseases in humans. Although cases exhibit generally increased homozygosity relative to controls, more detailed analysis indicates that this is largely due to a small subset of markers, each of which contributes a significant risk factor when homozygous. We conclude that heterozygosity at a minimum of five loci contributes ORs of up to 40, and that the most important loci vary depending on the type of pathogen.

There is currently a debate as to whether the benefits of heterozygosity accrue mainly through genome-wide effects (inbreeding) [38,39] or through individual balanced polymorphisms [13,14]. We found that inbreeding effects are either small or absent in this population. This is perhaps not surprising because, in contrast to some other populations such the Fulani [40] and some Arab communities [19], consanguineous marriages tend to be discouraged, with a preference for marriages between rather than within clans [41]. In contrast, five loci independently contribute significant risk factors, lending strong support to the local effects model. However, it should be remembered that human populations differ greatly in their structure and that, in contrast to most animals populations, some human populations actually favour consanguineous marriages [19,40,42]. In such populations a rather different pattern may well emerge.

To find several balanced polymorphisms in a relatively small study of just 134 markers is surprising, given how few have been identified previously in humans [22]. Two factors may contribute to this discrepancy. First, a large majority of genome scans focus on complex, non-infectious diseases, and these are likely to differ from infectious diseases mechanistically. Most heritable non-infectious diseases involve mutant alleles at one or more loci where function is removed or disrupted, and hence are mostly recessive. In contrast, the efficacy of immune-function genes is widely though to benefit from high diversity, a larger palette of alleles increasing the range of pathogen types that can be recognised, and therefore these loci tend naturally towards heterosis. Second, classical association studies tend to be applied to diseases that are known to run in families [43,44], and hence susceptibility will tend to have an appreciable additive component. As such, patterns where heterozygosity is important will tend to be overlooked because heterozygosity *per se *tends not to be heritable. Instead there is a strong focus on searching for associations between particular alleles and disease [43,45,46]. It will be interesting to see the extent to which future studies reveal a much higher prevalence of balancing selection, thereby supporting results from many non-human systems.

Our current study is relatively small-scale, with several of the smaller chromosomes being scored for only three or four markers. Consequently, there are large tracts of the genome where further loci could be located with the potential to contribute even further to genetic susceptibility, and implying that the five regions we identify are not the complete set of the loci that could potentially be identified in a larger study. This is surprising because the loci we have uncovered exhibit large individual and combined effect sizes, to the extent that mortality appears highly non-random. Moreover, it should be remembered that the overall risk factor combines both genetic susceptibility and variation in exposure. Unless exposure to pathogens is highly uniform, the impact of genetic factors will be even higher than we report and could rise further if our study has missed further contributory loci.

The effect sizes we report appear much larger than expected. Across the five loci identified as having highest impact, population attributable risk fractions (PARFs) all lie in the range 25–55%. PARFs provide an indication of the proportion of total risk that can be attributed to each genetic factor, given the local prevalence of exposure. Since the calculations assume overlapping effects, these do not sum to one. None the less, our analysis suggests that half or more of the observed deaths would probably not have occurred if the individuals concerned had been heterozygous for these loci, a figure that would surely be even higher if we had been able to genotype SNPs in the genes concerned rather than at linked microsatellites.

The idea that pathogens could play a major role in driving balancing selection at many different locations across the genome is reinforced by the difference we found between Gram negative and Gram positive bacteria. Immune defense mechanisms against Gram positive and Gram negative pathogens vary significantly [47,48], and while there may be some degree of overlap in genetic regulation of immunity to different classes of pathogens, the difference we find between Gram negative and Gram positive strains would help to explain why so many different regions appear to be involved.

## Conclusion

We believe our study is the first to apply to humans the sorts of analysis that commonly reveal single locus heterosis maintained by pathogens in natural populations. We reveal several discrete genomic locations where heterozygosity confers some degree of protection from lethal bacterial infection. Together these loci contribute a substantial risk factor that makes mortality from infection highly non-random. Our study has obvious implications for epidemiology and could lead to the development of simple tests for individuals who are most at risk from infection. High density SNP mapping is under way in order to identify relevant genes.

## Methods

Meningitis is defined by a positive cerebrospinal fluid culture. Neonatal sepsis is defined as bacteraemia or meningitis from day 0 to 59 of life. Malaria parasitemia was concurrently present in some cases and these are analysed as a separate class because malaria may have contributed to mortality.

### Control selection

Controls were selected at random from among a set of healthy subjects who had originally been selected from the community living near a case using the "spinning pencil" technique and individually matched to cases on age, sex and date of presentation to hospital in a case-control study of both surviving and fatal cases of bacteraemia. For ethical reasons, no controls were recruited among young infants (age <60 days). Cases and controls were restricted to the Mijikenda ethnic group indigenous to Coastal Kenya. The subset of controls selected for the present study was frequency-matched on age to cases in the present study. In all multi-variable logistic regression models age and administrative location of residence were included. Age was specified in six strata (0–5 m, 6–11 m. 12–23 m, 24–35 m, 36–59 m, 60–151 m) each of which contained between 13–19% of the observations. To control for ethnic diversity we stratified by administrative authority, the best form of 'address' we could obtain, yielding eight geographical locations each of which contained between 4–26% of the data. These partitions allow for some degree of geographic substructure and correspond loosely with seven long established sub-groups of the Mijikenda ethnic group, each of which has a different language, and who tend to live in geographically defined clusters.

### Standardized Observed Homozygosity



*S*_*OH *_is the standardized observed homozygosity for an individual genotyped for *i *loci. *N*_*hom *_is the number of homozygote genotypes in the individual concerned and *H*_*oi *_is the observed frequency of homozygotes the *i*^th ^locus scored in this individual, calculated across the full sample set.

### Population Attributable Risk Fractions

The PARFs were estimated as prev(OR-1)/(1+prev(OR-1) for each marker in the final model of all invasive bacterial disease deaths combined but, for simplicity, excluding the interaction terms. The prevalence of homozygosity in the population was estimated in the control population after standardizing on age to the known age-distribution of the population around the hospital. This was provided by the Kilifi Demographic Surveillance Study, which has conducted 2–3 household visits each year to enumerate the population in an area accommodating 230,000 people living closest to the hospital since 2000.

## Competing interests

The authors declare that they have no competing interests.

## Authors' contributions

EL conducted the genotyping, participated in the analysis and helped write the paper. WA helped conceive the study, led the analysis and wrote the paper. JB, IM, KM, NP and AS conducted the case control study and defined the clinical syndromes, JB and AS participated in the study design and interpretation of clinical data and AS further conducted the statistical analysis and helped write the paper. MS TRW CRN participated in study coordination, sample acquisition and processing, and interpretation of the data. AH initiated the genetic programme and directed the Oxford research activities.

## Pre-publication history

The pre-publication history for this paper can be accessed here:



## Supplementary Material

Additional file 1 **Names and chromosomal assignments of all microsatellites genotyped in this study.** Click here for file
