# Supplementary material for: Homozygosity and risk of childhood death due to invasive bacterial disease
Source: BMC Med Genet. 2009 Jun 12;10:55. doi: 10.1186/1471-2350-10-55 (PMC2714084; doi:10.1186/1471-2350-10-55)
Supplement: Additional file 1 — Names and chromosomal assignments of all microsatellites genotyped in this study. [file 1471-2350-10-55-S1.doc]

**Additional file 1**

| **marker** | **Chr** | **Het** | **marker** | **Het** | **Het** | **marker** | **Chr** | **Het** |
| --- | --- | --- | --- | --- | --- | --- | --- | --- |
| D1S199 | 1 | 0.76 | D5S433 | 5 | 0.71 | D13S171 | 13 | 0.71 |
| D1S255 | 1 | 0.77 | D5S1981 | 5 | 0.55 | D13S265 | 13 | 0.67 |
| D1S2726 | 1 | 0.74 | D5S408 | 5 | 0.68 | D14S261 | 14 | 0.52 |
| D1S2800 | 1 | 0.71 | D6S1610 | 6 | 0.76 | D14S275 | 14 | 0.59 |
| D1S2841 | 1 | 0.67 | D6S281 | 6 | 0.85 | D14S280 | 14 | 0.72 |
| D1S2868 | 1 | 0.65 | D6S289 | 6 | 0.74 | D14S288 | 14 | 0.61 |
| D1S2878 | 1 | 0.81 | D6S1574 | 6 | 0.75 | D14S292 | 14 | 0.69 |
| D1S2890 | 1 | 0.72 | D6S264 | 6 | 0.79 | D14S63 | 14 | 0.68 |
| D1S413 | 1 | 0.68 | D6S287 | 6 | 0.76 | D14S74 | 14 | 0.67 |
| D1S498 | 1 | 0.39 | D6S292 | 6 | 0.55 | D14S985 | 14 | 0.66 |
| D2S112 | 2 | 0.50 | D7S486 | 7 | 0.63 | D15S1007 | 15 | 0.57 |
| D2S117 | 2 | 0.76 | D7S493 | 7 | 0.43 | D15S120 | 15 | 0.53 |
| D2S125 | 2 | 0.93 | D7S519 | 7 | 0.61 | D15S128 | 15 | 0.49 |
| D2S142 | 2 | 0.74 | D7S531 | 7 | 0.02 | D16S3103 | 16 | 0.40 |
| D2S160 | 2 | 0.77 | D7S661 | 7 | 0.74 | D16S415 | 16 | 0.59 |
| D2S165 | 2 | 0.67 | D7S798 | 7 | 0.75 | D16S423 | 16 | 0.48 |
| D2S168 | 2 | 0.86 | D8S284 | 8 | 0.44 | D17S1852 | 17 | 0.70 |
| D2S2211 | 2 | 0.81 | D8S505 | 8 | 0.69 | D17S1868 | 17 | 0.66 |
| D2S2382 | 2 | 0.81 | D8S550 | 8 | 0.30 | D17S798 | 17 | 0.54 |
| D2S335 | 2 | 0.66 | D8S270 | 8 | 0.24 | D17S831 | 17 | 0.81 |
| D2S337 | 2 | 0.84 | D9S161 | 9 | 0.74 | D17S785 | 17 | 0.50 |
| D2S396 | 2 | 0.81 | D9S167 | 9 | 0.18 | D17S921 | 17 | 0.26 |
| D3S1271 | 3 | 0.80 | D9S175 | 9 | 0.81 | D17S928 | 17 | 0.49 |
| D3S1311 | 3 | 0.84 | D9S288 | 9 | 0.98 | D18S1102 | 18 | 0.39 |
| D3S1565 | 3 | 0.78 | D9S164 | 9 | 0.65 | D18S61 | 18 | 0.52 |
| D3S1614 | 3 | 0.74 | D9S285 | 9 | 0.59 | D18S70 | 18 | 0.75 |
| D3S1304 | 3 | 0.79 | D9S287 | 9 | 0.73 | D18S452 | 18 | 0.56 |
| D3S1569 | 3 | 0.90 | D10S192 | 10 | 0.43 | D18S53 | 18 | 0.54 |
| D3S1267 | 3 | 0.20 | D10S1653 | 10 | 0.60 | D18S59 | 18 | 0.55 |
| D3S1580 | 3 | 0.96 | D10S208 | 10 | 0.16 | D18S462 | 18 | 0.71 |
| D3S2338 | 3 | 0.77 | D10S212 | 10 | 0.54 | D18S64 | 18 | 0.28 |
| D4S392 | 4 | 0.81 | D10S189 | 10 | 0.41 | D19S210 | 19 | 0.53 |
| D4S405 | 4 | 0.78 | D10S537 | 10 | 0.67 | D19S221 | 19 | 0.46 |
| D4S1572 | 4 | 0.41 | D10S587 | 10 | 0.73 | D19S414 | 19 | 0.57 |
| D4S391 | 4 | 0.77 | D10S249 | 10 | 0.49 | D19S902 | 19 | 0.39 |
| D4S415 | 4 | 0.89 | D11S902 | 11 | 0.68 | D20S112 | 20 | 0.35 |
| D4S426 | 4 | 0.85 | D11S904 | 11 | 0.43 | D20S115 | 20 | 0.24 |
| D4S2964 | 4 | 0.66 | D11S905 | 11 | 0.53 | D20S117 | 20 | 0.45 |
| D4S402 | 4 | 0.70 | D11S987 | 11 | 0.61 | D20S171 | 20 | 0.59 |
| D4S403 | 4 | 0.95 | D11S1320 | 11 | 0.69 | D20S196 | 20 | 0.39 |
| D4S424 | 4 | 0.83 | D11S4151 | 11 | 0.61 | D20S107 | 20 | 0.35 |
| D5S400 | 5 | 0.69 | D11S901 | 11 | 0.85 | D21S266 | 21 | 0.41 |
| D5S419 | 5 | 0.82 | D12S310 | 12 | 0.74 | D22S274 | 22 | 0.49 |
| D5S422 | 5 | 0.85 | D12S352 | 12 | 0.59 | D22S420 | 22 | 0.38 |
| D5S424 | 5 | 0.59 | D13S158 | 12 | 0.49 |  |  |  |
